# Epigenetic-Genetic Chromosome Dosage Approach for Fetal Trisomy 21 Detection Using an Autosomal Genetic Reference Marker

**DOI:** 10.1371/journal.pone.0015244

**Published:** 2010-12-20

**Authors:** Yu K. Tong, Rossa W. K. Chiu, Ranjit Akolekar, Tak Y. Leung, Tze K. Lau, Kypros H. Nicolaides, Y. M. Dennis Lo

**Affiliations:** 1 Centre for Research into Circulating Fetal Nucleic Acids, Li Ka Shing Institute of Health Sciences, The Chinese University of Hong Kong, Prince of Wales Hospital, Shatin, Hong Kong Special Administrative Region, China; 2 Department of Chemical Pathology, The Chinese University of Hong Kong, Prince of Wales Hospital, Shatin, Hong Kong Special Administrative Region, China; 3 Harris Birthright Research Centre for Fetal Medicine, King's College Hospital, London, United Kingdom; 4 Department of Obstetrics and Gynaecology, The Chinese University of Hong Kong, Prince of Wales Hospital, Shatin, Hong Kong Special Administrative Region, China; King's College London, United Kingdom

## Abstract

**Background:**

The putative promoter of the *holocarboxylase synthetase* (*HLCS*) gene on chromosome 21 is hypermethylated in placental tissues and could be detected as a fetal-specific DNA marker in maternal plasma. Detection of fetal trisomy 21 (T21) has been demonstrated by an epigenetic-genetic chromosome dosage approach where the amount of hypermethylated *HLCS* in maternal plasma is normalized using a fetal genetic marker on the Y chromosome as a chromosome dosage reference marker. We explore if this method can be applied on both male and female fetuses with the use of a paternally-inherited fetal single nucleotide polymorphism (SNP) allele on a reference chromosome for chromosome dosage normalization.

**Methodology:**

We quantified hypermethylated *HLCS* molecules using methylation-sensitive restriction endonuclease digestion followed by real-time or digital PCR analyses. For chromosome dosage analysis, we compared the amount of digestion-resistant *HLCS* to that of a SNP allele (rs6636, a C/G SNP) that the fetus has inherited from the father but absent in the pregnant mother.

**Principal Findings:**

Using a fetal-specific SNP allele on a reference chromosome, we analyzed 20 euploid and nine T21 placental tissue samples. All samples with the fetal-specific C allele were correctly classified. One sample from each of the euploid and T21 groups were misclassified when the fetal-specific G allele was used as the reference marker. We then analyzed 33 euploid and 14 T21 maternal plasma samples. All but one sample from each of the euploid and T21 groups were correctly classified using the fetal-specific C allele, while correct classification was achieved for all samples using the fetal-specific G allele as the reference marker.

**Conclusions:**

As a proof-of-concept study, we have demonstrated that the epigenetic-genetic chromosome dosage approach can be applied to the prenatal diagnosis of trisomy 21 for both male and female fetuses.

## Introduction

The predominant reason for pregnant women to opt for prenatal diagnosis is the possibility of fetal chromosomal aneuploidies, in particular, trisomy 21 (T21, Down syndrome). The use of invasive procedures to obtain fetal genetic materials for a definitive diagnosis is associated with a finite risk of fetal loss [1]–[3]. Noninvasive screening tests by ultrasonography and maternal serum biochemical markers measure the epiphenomena rather than the actual chromosome dosage of the fetus [4], [5]. Despite a reasonably useful sensitivity and specificity profile, the fact that prenatal screening tests are typically usable at a relatively narrow gestational window and the need to combine multiple markers have prompted workers to explore new approaches for noninvasive prenatal diagnosis.

The discovery of cell-free fetal DNA in maternal plasma in 1997 has opened up new avenues for prenatal diagnosis [6], [7]. Fetal DNA exists as a minor fraction of background maternal DNA in maternal plasma [8]. Nonetheless, detection of genetic markers that the fetus has inherited from the father but are absent in the pregnant mother have been implemented into clinical use. Such qualitative assessments include the noninvasive determination of fetal rhesus D status [9], [10] and exclusion of sex-linked disorders [11]. However, detection of fetal chromosomal aneuploidies requires precise quantitative measurements of the nucleic acid molecules, and it has represented a challenge in the field.

Direct detection of fetal trisomy by allelic ratio analysis of single nucleotide polymorphisms (SNPs) present in fetal-specific nucleic acid markers in maternal plasma has been described with the RNA-SNP approach [12]–[15] and epigenetic allelic ratio approach [16]. The allelic ratio strategy targets a subset of nucleic acids molecules that are representative of the fetal genome but present in maternal plasma, e.g., placental mRNA transcripts [17]–[19] or DNA molecules bearing a placenta-specific methylation signature [20]–[24]. Since the fetus has to be heterozygous for the SNP to be informative for allelic ratio analysis, the use of multiple markers is required to increase the population coverage. However, it would be time-consuming to develop such a list of markers because a SNP has to be present within each fetal-specific marker in order to use this approach.

Recently, noninvasive prenatal detection of fetal chromosomal aneuploidies has been demonstrated with the highly precise massively parallel genomic sequencing method [25]–[27]. By counting an extremely large number of DNA molecules, e.g., millions of molecules, in a sample, the slight increment in genomic representation from a trisomic chromosome derived from a trisomic fetus could be detected [28]. Nonetheless, this is an expensive approach with a relatively low throughput.

We have recently proposed a novel epigenetic-genetic chromosome dosage approach for fetal trisomy 21 detection using a fetal epigenetic marker, the putative promoter of the *holocarboxylase synthetase* (*HLCS*) gene (NM_000411) on chromosome 21, and a fetal genetic marker, the *zinc finger protein, Y-linked* (*ZFY*) gene (NM_003411) present on chromosome Y [29]. The rationale of this approach is that the dosage of chromosome 21 in the genome of the fetus is inferred by analyzing the amount of *HLCS* molecules in maternal plasma and to normalize it to a genetic marker that is fetal-specific in maternal plasma. To demonstrate that this method can be used for both male and female fetuses, we have explored the use of a paternally-inherited fetal SNP allele on a reference chromosome to serve as the baseline for epigenetic-genetic chromosome 21 dosage determination in place of the chromosome Y marker.

## Materials and Methods

### Ethics statement

This study was conducted according to the principles expressed in the Declaration of Helsinki. Ethics approval was obtained from the Joint Chinese University of Hong Kong – New Territories East Cluster Clinical Research Ethical Committee and the King's College Hospital Ethics Committee. All patients provided written informed consent for the collection of samples and subsequent analysis.

### Study Participants

Women with euploid and T21 pregnancies who attended the Department of Obstetrics and Gynaecology, Prince of Wales Hospital and the King's College Hospital, UK were recruited between September 2004 and March 2010. Informed consent was obtained from individuals who joined the study.

Chorionic villus samples (CVS) and amniotic fluid samples were collected during conventional prenatal diagnosis sessions (gestational age: 12 to 18 weeks). Placental tissue samples were collected from euploid third-trimester pregnancies after delivery (gestational age: 37 to 40 weeks) and from T21 pregnancies after termination of pregnancy (gestational age: 12 to 21 weeks). The chromosome status of each T21 case was confirmed by full karyotyping. Maternal peripheral blood samples (12 mL in EDTA tubes) were collected from all subjects.

### Processing of Blood and Tissue Samples

Peripheral blood samples were processed by a double centrifugation protocol as previously described [30]. The blood cell portion was recentrifuged at 2,500 *g*, and any residual plasma was removed. All processed blood samples were stored at −80°C until DNA extraction. DNA from the peripheral blood cells and that from maternal plasma was extracted with the blood and body fluid protocol of the QIAamp DNA Blood Mini Kit and the QIAamp DSP DNA Blood Mini Kit, respectively (Qiagen).

Amniotic fluid samples were collected and stored at 4°C until use. DNA from amniotic fluid was extracted with the blood and body fluid protocol of the QIAamp DNA Blood Mini Kit (Qiagen).

CVS and placental tissues were collected and stored at −80°C until use. DNA from the CVS and placentas was extracted with the QIAamp DNA Mini Kit (Qiagen) according to the manufacturer's tissue protocol.

Plasma and CVS samples collected at the King's College Hospital were processed and shipped on dry ice to Hong Kong for molecular analysis.

### Genotyping of the rs6636 SNP

Genotyping of the rs6636 SNP for the fetus (CVS, placental tissue or amniotic fluid) and the mother (maternal blood cells) was performed using a primer extension protocol as previously described [16]. The sequences of the primers for genotyping are listed in Table S1. Informative cases, defined as one in which the fetus was heterozygous and the mother was homozygous for the rs6636 SNP, were subjected to the epigenetic-genetic chromosome dosage analysis.

### Chromosome Dosage Analysis

**Figure 1:**
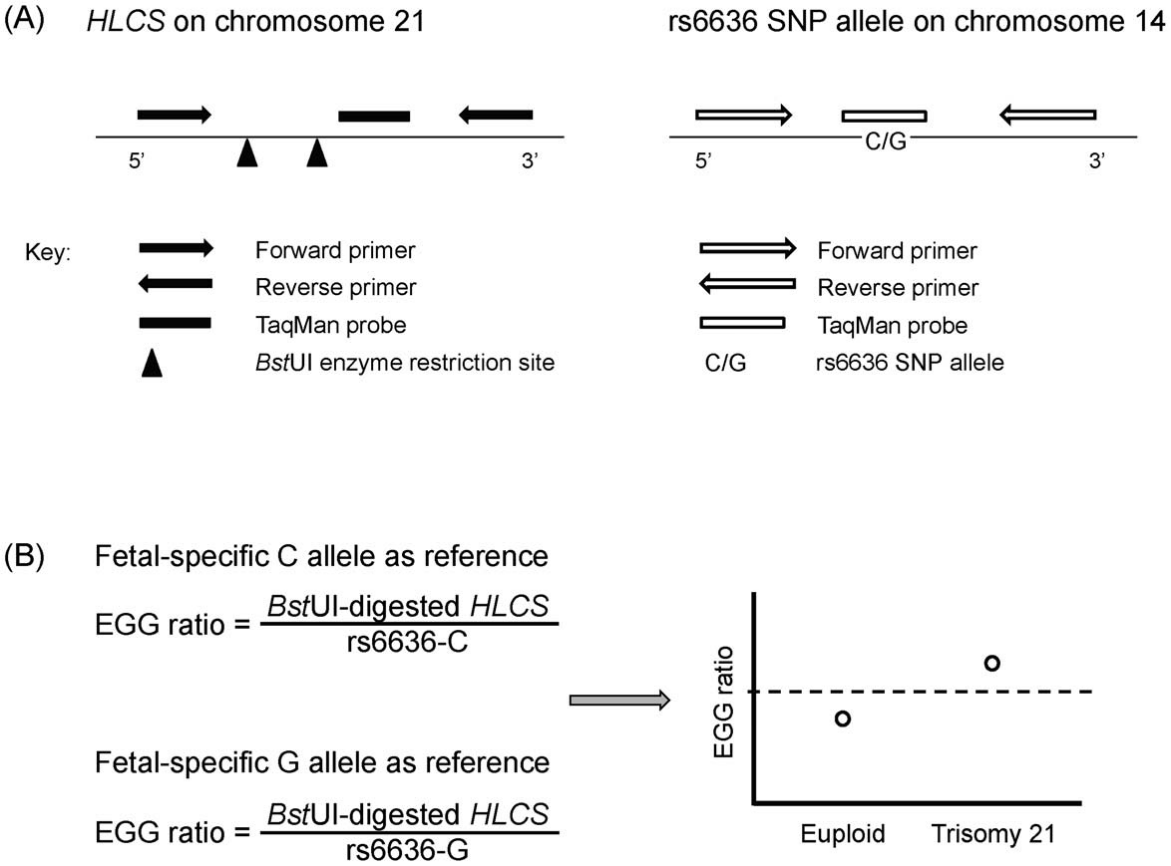
Schematic diagram of the *HLCS* and rs6636 SNP assays with the interpretation of the epigenetic-genetic (EGG) ratio. (A) Left, the *HLCS* assay located in a CpG island on chr21:38352994-38353281 (reverse strand) of the February 2009 (hg19) assembly of the human genome using the UCSC Genome Browser. Right, the rs6636 SNP assay located on chr14:77801610-77801705 (hg19). The relative positions of the primers, probes, *Bst*UI enzyme restriction sites and SNP of each assay are indicated. (B) EGG ratio can be measured by using the paternally-inherited, fetal-specific SNP allele as reference. An elevation of the EGG ratio is observed in T21 in relation to the cutoff value (illustrated by the dotted line) established from the euploid samples.

The chromosome 21 and the reference markers in placental tissue and maternal plasma DNA samples were quantified by real-time quantitative polymerase chain reaction (qPCR) and digital PCR [31], respectively. Chromosome dosage analysis was performed by comparing the amount of hypermethylated *HLCS* to that of a SNP allele (rs6636, a C/G SNP) that the fetus has inherited from the father but absent in the pregnant mother. SNP rs6636 is located within the *transmembrane emp24 protein transport domain containing 8* (*TMED8*) gene (AK095650) on chromosome 14 with an average heterozygosity of 0.452+/−0.147 (dbSNP build 131). rs6636 is one of the SNPs that we have previously analyzed using mass spectrometry [32]. The rs6636 SNP assays are denoted as rs6636-C/G SNP assays in this manuscript (Figure 1).

#### Methylation-sensitive restriction endonuclease (MSRE) digestion

A methylation-sensitive restriction endonuclease, *Bst*UI (New England Biolabs), was used to digest the hypomethylated DNA. Extracted DNA was digested with the *Bst*UI enzyme at 60°C for 16 hours. For CVS, placental tissues and maternal and normal control blood cells, 40 U of *Bst*UI enzyme was used to digest 100 ng of DNA for the PCR assays. A mock-digested aliquot was included as the digestion control. For mock-digestion, an equal amount of DNA was subjected to the same digestion condition without the addition of enzyme. For the plasma samples, 20 U to 60 U of the *Bst*UI enzyme was used to digest the DNA from 1.6–5.2 mL plasma.

#### Assay design and reaction conditions for real-time quantitative PCR (qPCR) analysis

Real-time qPCR analysis was performed for the *HLCS* and rs6636 loci. The sequences of the primers and probes are listed in Table 1. Assays for the *HLCS*, rs6636-C allele and rs6636-G allele were all performed in a monoplex format. Within the PCR amplicon of the *HLCS* locus, there were two *Bst*UI enzyme recognition sites. In contrast, the rs6636 SNP assays did not contain any *Bst*UI enzyme recognition sites. A schematic illustration of the assay design is shown in Figure 1.

**Table 1:** Oligonucleotide sequences for the *HLCS*, rs6636-C/G SNP and *beta-actin* PCR assays.

*Assay*	*Oligonucleotide*	*Sequences (5′ to 3′)*	*PCR amplicon length (bp)*
*HLCS*	Forward primer	CCGTGTGGCCAGAGGTG	96
	Reverse primer	AAAGGGCCAGGTCGGGA	
	TaqMan probe (FAM)	FAM-AGGATTTGGGGCTGCGC(MGB)^a^	
	TaqMan probe (VIC)	VIC-AGGATTTGGGGCTGCGC(MGB)^b^	
rs6636-C/G	Forward primer	TGGTAAGACTCTTAGAAATCACAGATGTT	96
	Reverse primer	GTATCCCAACTAATCATTTATTATGGTCA	
	TaqMan probe_ rs6636-C	FAM-CCCCTATCATGAGAAAT(MGB)	
	TaqMan probe_ rs6636-G	VIC-CCCCTATGATGAGAAAT(MGB)	
*Beta-actin*	Forward primer	CCACCACCGCCGAGAC	96
	Reverse primer	TGGCCGGGCTTACCTGG	
	TaqMan probe	FAM-AGCACAGAGCCTCGCC(MGB)	

a FAM  = 6-carboxyfluorescein, MGB  =  minor-groove binding.

b VIC  =  Applied Biosystems® proprietary dye.

Each reaction was set up as a 25 µL mixture at a final concentration of 1× TaqMan® Universal PCR Master Mix (Applied Biosystems), 100 nM TaqMan® probe (Applied Biosystems), and 300 nM of each of the forward and reverse primers (Integrated DNA Technologies) with 25 ng DNA input. The reaction was initiated at 50°C for 2 min and continued at 95°C for 10 min and followed by 40 cycles of 95°C for 15 s and 60°C for 1 min. The experiments were carried out on the 7300 Real-time PCR System (Applied Biosystems) and the fluorescence data were collected and analyzed by the SDS v1.3.0 software (Applied Biosystems). All reactions were run in duplicates with the mean quantity taken. A calibration curve was constructed by serially-diluted genomic DNA extracted from adult male blood cells with concentration ranging from 10,000 genome equivalents (GE) to 3 GE per reaction.

#### Assay design and reaction conditions for the digital PCR analysis

The same oligonucleotide sequences for real-time PCR analysis were used for the *HLCS* and rs6636-C/G SNP digital PCR analyses (Table 1). Here, the *HLCS* assay was performed as a duplex reaction with each of the rs6636-C/G SNP assays. The fluorescent probes were labeled as *HLCS*(VIC) duplex with rs6636-C(FAM) and *HLCS*(FAM) duplex with rs6636-G(VIC).

The basis of the digital PCR analysis have been described previously [13], [33]. The total reaction volume was 5 µL per well in a 384-well plate at a final concentration of 1× TaqMan® Universal PCR Master Mix (Applied Biosystems), 100 nM TaqMan® probe (Applied Biosystems), and 300 nM of each of the forward and reverse primers (Integrated DNA Technologies). The reaction was initiated at 50°C for 2 min and continued at 95°C for 10 min and followed by 50 cycles of 95°C for 15 s and 60°C for 1 min. The experiments were carried out on the 7900HT Sequence Detection System (Applied Biosystems) in a 384-well format, and the fluorescence data were collected by the “Absolute Quantification” application of SDS 2.3 software (Applied Biosystems).

#### Specificity of the rs6636-C/G SNP assay

Genomic DNA extracted from placental tissues with known rs6636 genotypes were subjected to the *HLCS* and rs6636 duplex assays. Samples that were homozygous for one allele were tested with the rs6636-C/G SNP assay for the other allele. A sample homozygous for the C allele should not show any signals for the rs6636 assay detecting the G allele, and *vice versa*.

### *Beta*-*actin* assay as a digestion control

A previously-described *beta-actin* region which is unmethylated in both the placenta and maternal blood cells was used to determine the efficiency of *Bst*UI digestion [34]. The *beta-actin* assay was modified to contain two *Bst*UI enzyme recognition sites to match the number of *Bst*UI enzyme recognition sites of the current *HLCS* assay. The sequences of the primers and probes are listed in Table 1. The same sequences were used for both the real-time qPCR and digital PCR analyses.

### Statistical analysis

Statistical analyses were performed using the SigmaStat 3.5 software (SPSS).

## Results

### Chromosome dosage analysis by conventional real-time qPCR

The *HLCS* and rs6636-C/G SNP assays were applied to a total of 20 euploid and nine T21 placental tissue samples with the heterozygous C/G genotype. Six euploid and four T21 samples were analyzed by using the *HLCS* to rs6636-C ratio, and the remaining 14 euploid and five T21 samples were analyzed by the *HLCS* to rs6636-G ratio. A calibration curve constructed by serially-diluted genomic DNA extracted from adult male blood cells with concentration ranging from 10,000 GE to 3 GE per reaction was used to determine the absolute copy number of the two loci in the tested samples.

All three assays were optimized to have similar efficiencies, as indicated by the slopes and *y*-intercepts of the calibration curves. The slopes of the calibration curves of the *HLCS*, rs6636-C and rs6636-G assays were −3.71, −3.70 and −3.62, respectively, whereas the *y*-intercepts showed threshold cycle values of 39.62, 39.79 and 42.04, respectively.

#### *HLCS* to rs6636-C ratio in placental DNA samples

**Figure 2:**
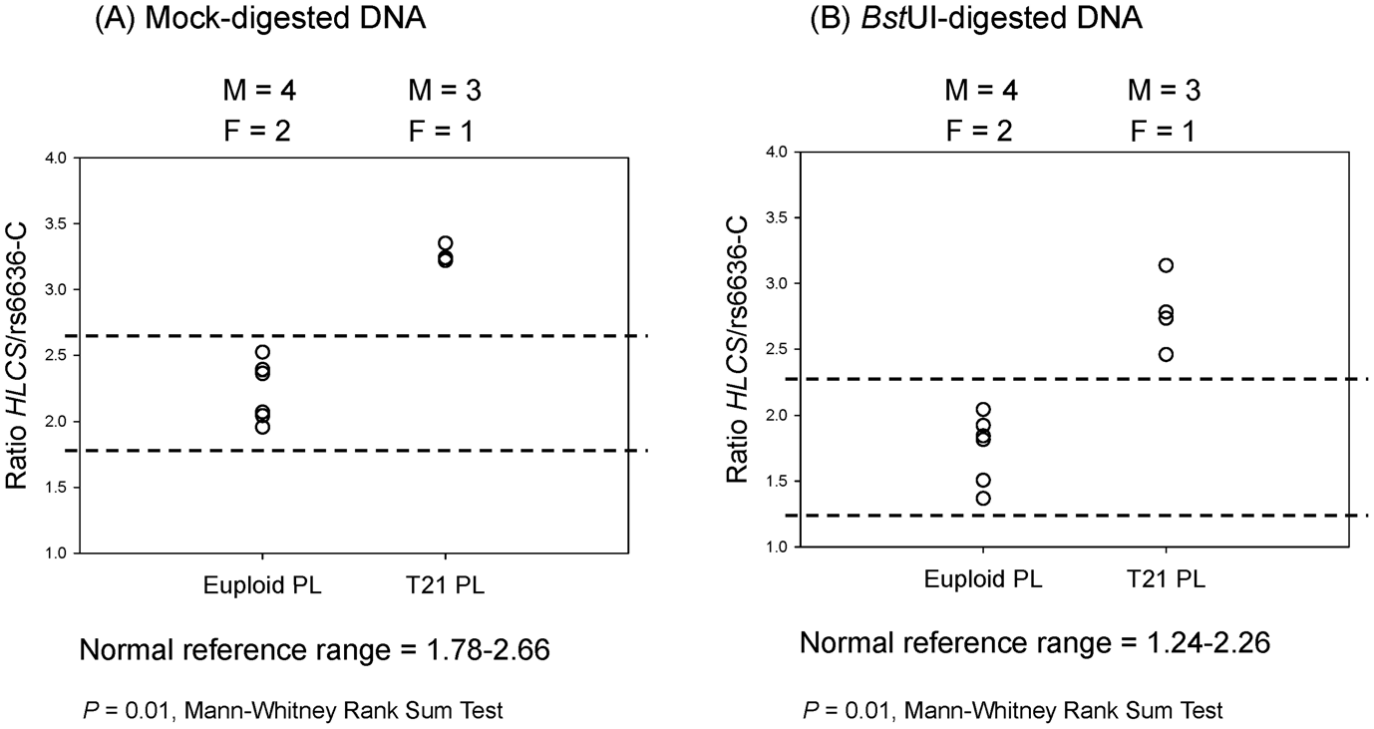
Chromosome 21 dosage using rs6636-C allele as reference in euploid and T21 placental DNA samples. Ratio of (A) *HLCS* to rs6636*-*C with mock digestion, and (B) *HLCS* to rs6636*-*C with *Bst*UI digestion. The normal reference range is depicted by dotted lines. M, male fetus; F, female fetus. PL, placenta.

The *HLCS* to rs6636-C ratio was first determined in mock-digested placental DNA samples. These included four male and two female fetuses for the euploid, and three male and one female fetuses for the T21 samples. A normal reference range, defined as the mean *HLCS* to rs6636-C ratio ± 1.96SD, was calculated from the six euploid samples as 1.78–2.66. All four T21 samples showed a higher ratio than the normal reference range (Figure 2A).

The same samples were then subjected to *Bst*UI restriction enzyme digestion before real-time PCR analysis. While the unmethylated *HLCS* molecules would be digested by *Bst*UI enzyme treatment, leaving only the digestion-resistant, hypermethylated *HLCS* molecules for PCR detection, the rs6636 molecules would remain intact as there was no *Bst*UI enzyme recognition site within the rs6636 PCR amplicon. A normal reference range was calculated as 1.24–2.26. All samples were correctly classified (Figure 2B).

In both the mock and *Bst*UI digestion experiments, a placental DNA sample, N2541, with the homozygous GG genotype for the rs6636 SNP was included for testing the assay specificity. There was no signal when the rs6636 assay for detecting the C allele was applied (Table 2).

**Table 2:** Specificity of the rs6636-C/G SNP assays by real-time qPCR.

Assays: *HLCS* and rs6636-C				
Treatment	Sample	rs6636 genotype	Copies per reaction	
			*HLCS*	rs6636-C
Mock digestion	N2541	GG	4226	0
*Bst*UI-digestion	N2541	GG	4402	0

Assays: *HLCS* and rs6636-G				
Treatment	Sample	rs6636 genotype	Copies per reaction	
			*HLCS*	rs6636-G
Mock digestion	N2582	CC	2177	0
*Bst*UI-digestion	N2582	CC	1439	0

#### *HLCS* to rs6636-G ratio in placental DNA samples

**Figure 3:**
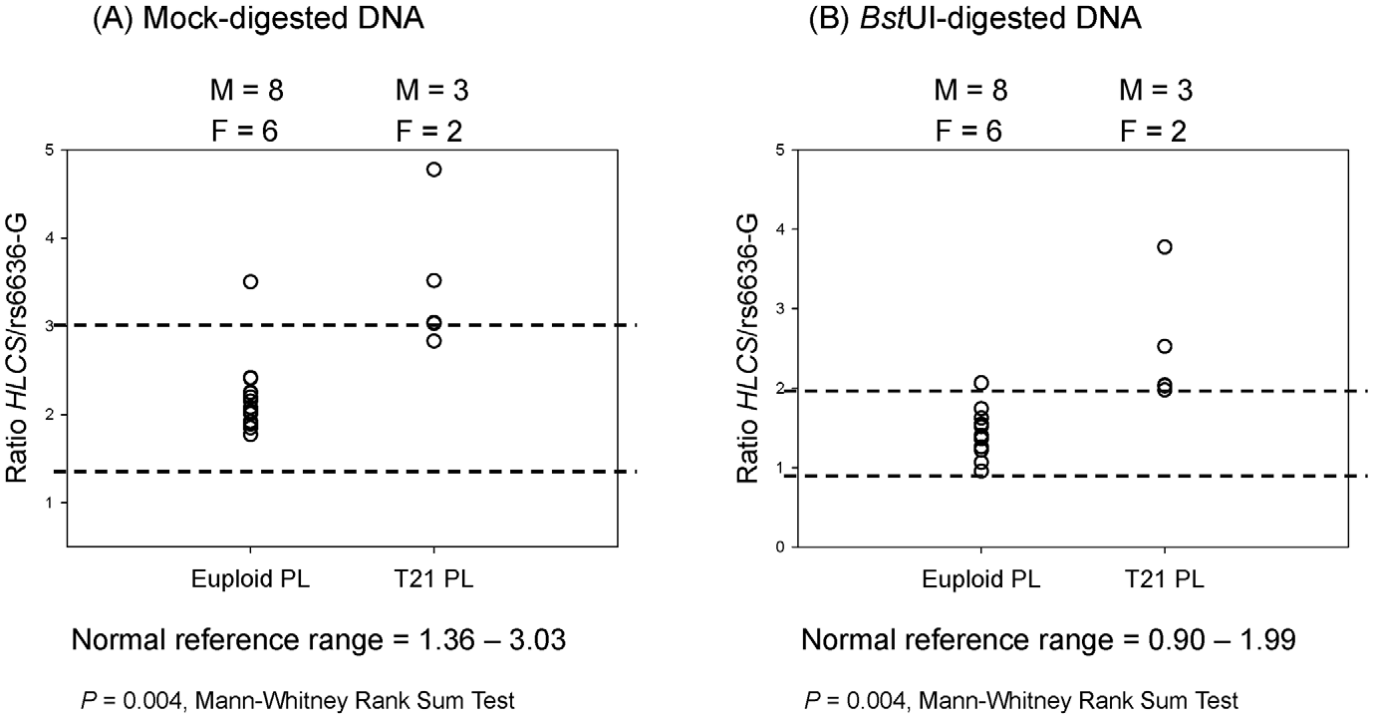
Chromosome 21 dosage using rs6636-G allele as reference in euploid and T21 placental DNA samples. Ratio of (A) *HLCS* to rs6636*-*G with mock digestion, and (B) *HLCS* to rs6636*-*G with *Bst*UI digestion. The normal reference range is depicted by dotted lines. M, male fetus; F, female fetus. PL, placenta.

The *HLCS* to rs6636-G ratio was first determined in mock-digested placental DNA samples. A normal reference range was calculated from the 14 euploid samples as 1.36–3.03. One sample from each of the euploid and T21 groups was misclassified (Figure 3A). Among these samples, the euploid pregnancies consisted of eight male and six female fetuses, while the T21 pregnancies had three male and two female fetuses.

The same samples were then subjected to *Bst*UI restriction enzyme digestion before real-time PCR analysis. A normal reference range was calculated as 0.90–1.99. One sample from each of the euploid and T21 groups was misclassified (Figure 3B). These are not the same samples as the misclassified ones in the mock digestion experiment.

In both the mock and *Bst*UI digestion experiments, a placental DNA sample, N2582, with the homozygous CC genotype for the rs6636 SNP was included for testing the assay specificity. There was no signal when the rs6636 assay for detecting the G allele was applied (Table 2).

#### *Beta*-*actin* as a digestion control

The *Bst*UI enzyme digestion efficiency was evaluated by applying the *beta*-*actin* assay to the same mock- and *Bst*UI-digested placental DNA samples as those used for *HLCS* to rs6636 chromosome dosage analysis. The same calibration standard was used to quantify the amount of *beta-actin* sequences in the samples. The slope of the calibration curve was −3.41, whereas the *y*-intercept showed threshold cycle value of 37.97. Comparing the values of the corresponding pairs of digested and mock-digested samples, over 96% of the DNA was digested in all tested samples (Tables S2 and S3).

### Chromosome dosage analysis by digital PCR

#### Specificity of the rs6636-C/G SNP assay

Placental DNA samples of the heterozygous C/G, homozygous C/C and homozygous G/G genotypes were tested with the *HLCS* and rs6636 duplex assay to ascertain the specificity for detecting either one of the SNP alleles. Two samples were used for each of the three groups. A sample homozygous for the C allele should not show any signals for the rs6636 assay detecting the G allele, and *vice versa*.

The number of wells that were positive for each of the target loci was counted. The total number of molecules distributed into the 384-well plate followed the Poisson distribution, and was corrected with the following equation: Target  =  -ln [*E*/*N*] × *N*, where Target is the Poisson-corrected counts of the target molecules, *E* is the number of negative (empty) wells, and *N* is the total number of digital PCRs in the experiment.

By applying the *HLCS* and rs6636-C duplex assay, placental DNA samples of the heterozygous C/G and homozygous C/C genotypes showed positive wells for both loci, whereas samples of the homozygous G/G genotypes were only positive for the *HLCS* locus, and no detectable signal was observed for the rs6636-C allele (Table 3).

**Table 3:** Specificity of the rs6636-C/G SNP assays by digital PCR.

Assays: duplex *HLCS* and rs6636-C				
Sample	rs6636 genotype	Copies/run*		No. of PCR wells analyzed
		*HLCS*	rs6636-C	
Placenta 1	CG	41	29	192
Placenta 2	CG	95	46	192
Placenta 3	CC	76	87	192
Placenta 4	CC	28	20	192
Placenta 5	GG	39	0	192
Placenta 6	GG	13	0	192

Assays: duplex *HLCS* and rs6636-G				
Sample	rs6636 genotype	Copies/run		No. of PCR wells analyzed
		*HLCS*	rs6636-G	
Placenta 1	CG	82	37	192
Placenta 2	CG	76	40	192
Placenta 3	CC	192	0	384
Placenta 4	CC	83	0	384
Placenta 5	GG	51	53	192
Placenta 6	GG	45	47	192

*The output for digital PCR is an “all-or-nothing” readout of individual amplification. The numbers of positive wells were counted for each target. With reference to the number of PCR wells analyzed, the copy numbers were corrected for the Poisson distribution using the formula shown in the Results section.

By applying the *HLCS* and rs6636-G duplex assay, placental DNA samples of the heterozygous C/G and homozygous G/G genotypes showed positive wells for both loci, whereas samples of homozygous C/C were only positive for the *HLCS* locus, and no detectable signal was observed for the rs6636-G allele (Table 3).

The results showed that the rs6636-C/G SNP assays were specific in detecting the individual SNP alleles. The specificity was conferred by the allele-specific probes labeled with difference fluorescent dyes.

#### *HLCS* to rs6636-C ratio in maternal plasma DNA samples

Maternal plasma DNA analysis was performed by comparing the ratio of digestion-resistant *HLCS* to fetal-specific rs6636 SNP allele in informative samples. An informative sample was defined as one in which the fetus was heterozygous and the mother was homozygous, being the rs6636-C/G SNP in the current example. The allele that the fetus had inherited from the father, and absent in the maternal genome, was used as the reference baseline for chromosome 21 dosage determination.

The *HLCS* and rs6636-C duplex assay was applied to a total of 18 euploid and six T21 pregnancies. In these samples, the fetal and maternal genotypes for the rs6636 SNP were C/G and G/G, respectively. The fetal-specific SNP allele was the C allele. Maternal plasma DNA samples were analyzed by digital PCR after *Bst*UI enzyme digestion. The total number of positive wells was used to calculate the ratio of *HLCS* to rs6636-C allele in each sample.

**Figure 4:**
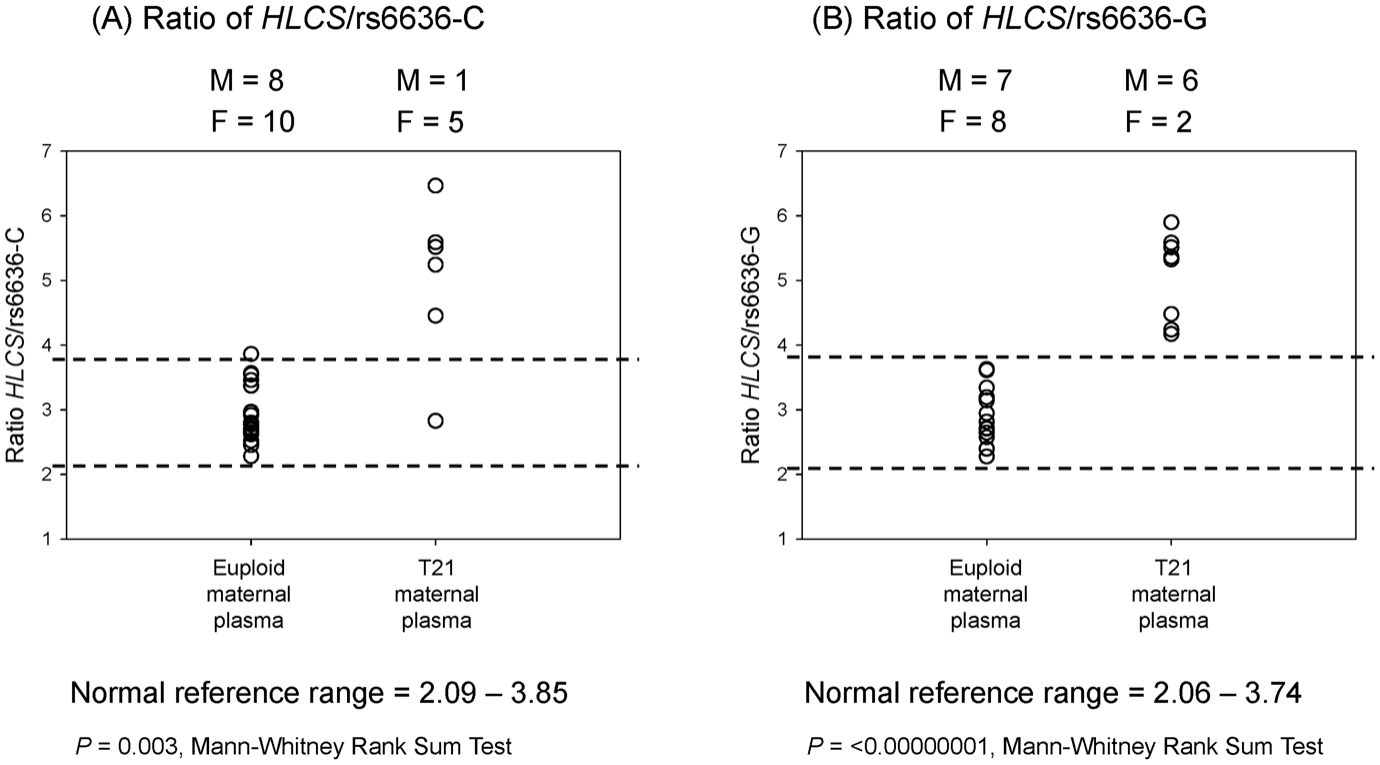
Chromosome 21 dosage determination in *Bst*UI-digested, euploid and trisomy 21 maternal plasma DNA samples. Ratio of (A) hypermethylated *HLCS* to rs6636*-*C allele, and (B) hypermethylated *HLCS* to rs6636*-*G allele. The normal reference range is depicted by dotted lines. M, male fetus; F, female fetus.

A normal reference range of 2.09–3.85 was calculated from the euploid maternal plasma samples. Nine, five and four samples were collected from the third, second- and first-trimester pregnancies, respectively. Among these 18 euploid samples, eight were from pregnancies carrying a male fetus, and ten from those carrying a female fetus with no gestational bias. The *HLCS* to rs6636-C allele ratios of all but one euploid sample fell within the reference range. A total of six T21 samples (one male and five female fetuses) were analyzed with the EGG approach, all but one sample had a ratio greater than the upper limit (Figure 4A).

#### *HLCS* to rs6636-G ratio in maternal plasma DNA samples

In the other group of informative samples, the fetal and maternal genotypes for the rs6636 SNP were C/G and C/C, respectively. The fetal-specific SNP allele was the G allele. The *HLCS* and rs6636-G duplex digital PCR assay was applied to *Bst*UI-digested maternal plasma DNA samples from 15 euploid pregnancies and eight T21 pregnancy. The total number of positive wells was used to calculate the ratio of *HLCS* to rs6636-G allele in each sample.

A normal reference range of 2.06–3.74 was calculated from euploid maternal plasma samples. Among the 15 euploid samples, five, six and four cases were collected from the third-, second- and first-trimester pregnancies, respectively. There were seven pregnancies with a male fetus, and eight with a female fetus. The *HLCS* to rs6636-G allele ratios of all euploid samples fell within the reference range. A total of eight T21 samples (six male and two female fetuses) were analyzed with correct classification for all samples (Figure 4B).

#### *Beta*-*actin* as a digestion control

The *Bst*UI enzyme digestion efficiency was evaluated by applying the *beta*-*actin* digital PCR assay to the same *Bst*UI-digested maternal plasma DNA samples as those used for the *HLCS* to rs6636 chromosome dosage analysis. An aliquot of the digested DNA samples (1/50 of the total digestion mixture) was confirmed to show no positive well for the *beta*-*actin* assay before being subjected to chromosome dosage analysis. If the results showed any positive digital PCR well, we would re-digest the plasma DNA to ensure that no control signal of *beta*-*actin* was detected.

## Discussion

We have recently demonstrated in principle that the EGG approach was a feasible method for fetal chromosome dosage analysis in maternal plasma DNA samples [29]. The hypermethylated *HLCS* locus was used as the epigenetic component, which represented a class of fetal-specific molecules, and the *ZFY* locus was a genetic marker which was specific to a male fetus. By comparing the ratio between the fetal-specific epigenetic marker on chromosome 21 and the fetal-specific genetic marker on a reference chromosome, the chromosome 21 dosage could be deduced.

In this paper, we further demonstrated that the epigenetic-genetic chromosome dosage approach can be applied to the prenatal diagnosis of trisomy 21 using a fetal-specific SNP allele as a genetic reference baseline in place of a chromosome Y marker derived from a male fetus. Here, SNP rs6636 located on chromosome 14 was used as an example. By comparing the ratio between the hypermethylated *HLCS* and the fetal-specific rs6636 SNP allele, the chromosome 21 dosage could be deduced in the majority of samples analyzed.

During the past few years, several methods have been proposed for the direct detection of fetal chromosomal aneuploidies by maternal plasma analysis. The earliest reports relied on measuring the allelic ratios of SNPs present in fetal-specific nucleic acid markers [12], [16]. Analysis of the allelic ratio approach requires the discrimination of an allelic ratio of 1∶1 in a euploid and 2∶1 in a trisomy fetus within a single locus. However, this approach is only applicable to fetuses who are heterozygous for the analyzed SNP located within the fetal-specific marker, thus limiting its population coverage. The EGG approach described in the present study also involves the measurement of an increase in the chromosome dosage in trisomy versus euploid pregnancies. However, it bypasses the requirement of the allelic ratio approach for both the fetal-specific marker and the SNP to be present in the same locus [29]. Since the number of fetal DNA molecules in maternal plasma is limited, identification of multiple markers to perform multiplex PCR analysis is suggested to increase the number of molecules that can be counted and hence improving the precision for the EGG ratio estimation. The quantitative precision of single molecule counting also opens up another class of methods for fetal trisomy detection that is independent of fetal-specific markers. These methods allow the detection of small quantitative perturbations in the genomic representation of plasma DNA. Examples of such approaches are those based on relative chromosome dosage analysis by digital PCR [13], and more recently, massively parallel genomic sequencing [25], [26]. The latter approach, in particular, appears to be very sensitive and specific, is limited by a high cost and relatively low throughput in the present laboratory settings.

Concerning the technical issues on the current development of EGG analysis, results from placental DNA analysis showed that one sample from each of the euploid and T21 groups were misclassified. Imprecision could be introduced by the use of monoplex conventional real-time PCR to measure the copy number for the *HLCS* and SNP loci to determine the epigenetic-genetic ratio. In light of this concern, we optimized the maternal plasma DNA analysis using duplex digital PCR assays. Despite a low concentration of fetal DNA in maternal plasma (typically, samples from early gestation had fewer than 50 copies of the paternally-inherited fetal SNP allele for analysis), only two samples were misclassified in a total of 47 maternal plasma DNA samples. Another technical issue was related to the choice of the digital PCR platform. The microfluidics digital PCR platform that we used previously contained a dead volume of about 50% [33]. The dead volume refers to the amount of reaction mixture that could not reach the partitioned PCR chambers due to the design of the system. As a result, half of the input DNA molecules would not be analyzed. Since the concentration of fetal DNA in maternal plasma is low, we switched to the more labor-intensive 384-well plate format for digital PCR analysis. We directly pipetted the template-containing reaction mixture into the PCR wells and hence the total volume of the reaction mixture could be analyzed. It is believed that an improved microfluidics digital PCR platform without dead volume would enhance the efficiency of the epigenetic-genetic chromosome dosage analysis.

In conclusion, we have shown that by using an informative SNP allele as the genetic reference baseline, the EGG chromosome dosage approach could be applied to the prenatal diagnosis of trisomy 21 for both male and female fetuses. Furthermore, development of a panel of genetic reference markers would allow broad population coverage of this approach. As previously described [32], one could first test a maternal buffy coat sample, which comprises mostly of maternal DNA, to ascertain the maternal genotypes for the panel of SNPs. For SNPs for which the mother is shown to be homozygous, one can then aim to detect the allele that is not represented in the maternal genotype in maternal plasma. If the plasma sample is positive for the non-maternal allele, it suggests that the fetus has inherited that allele from the father. The quantification of that paternally inherited fetal-specific allele in maternal plasma could then be used as the reference for gene dosage assessment using the epigenetic-genetic approach. Finally, the use of epigenetic markers for the other chromosomes involved in other aneuploidies important for prenatal testing, e.g. chromosomes 18 and 13, would further expand the clinical utility of the EGG approach.

## Supporting Information

Table S1 Oligonucleotide sequences for the rs6636-C/G SNP genotyping.
(DOC)

Table S2 Digestion efficiency evaluated by *beta*-*actin* real-time qPCR on samples for *HLCS* and rs6636-C analysis.
(DOC)

Table S3 Digestion efficiency evaluated by *beta*-*actin* real-time qPCR on samples for *HLCS* and s6636-G analysis.
(DOC)
