# Supplementary material for: Epigenetic-Genetic Chromosome Dosage Approach for Fetal Trisomy 21 Detection Using an Autosomal Genetic Reference Marker
Source: PLoS One. 2010 Dec 20;5(12):e15244. doi: 10.1371/journal.pone.0015244 (PMC3004793; doi:10.1371/journal.pone.0015244)
Supplement: Table S1 — Oligonucleotide sequences for the rs6636-C/G SNP genotyping. (DOC) [file pone.0015244.s001.doc]

**Table S1.** Oligonucleotide sequences for the rs6636-C/G SNP genotyping.

| *Assay* | *Oligonucleotide* | *Sequences (5' to 3')* |
| --- | --- | --- |
| rs6636-C/G genotyping | Forward primer | **ACGTTGGATG**GGTCAGCTTGTTTTACATGTC^a^ |
|  | Reverse primer | **ACGTTGGATG**GCAGGACACAAGAATTAAAG |
|  | Extension primer | AGCTTGTTTTACATGTCCCCTAT |

^a^ Bold nucloetides indicate the 10-mer tags added to the 5’ end of the primers in such a way that the masses of the primers would fall out of the analytic range of the mass spectrometry.
