# Supplementary material for: Epigenetic-Genetic Chromosome Dosage Approach for Fetal Trisomy 21 Detection Using an Autosomal Genetic Reference Marker
Source: PLoS One. 2010 Dec 20;5(12):e15244. doi: 10.1371/journal.pone.0015244 (PMC3004793; doi:10.1371/journal.pone.0015244)
Supplement: Table S2 — Digestion efficiency evaluated by beta-actin real-time qPCR on samples for HLCS and rs6636-C analysis. (DOC) [file pone.0015244.s002.doc]

**Table S2.** Digestion efficiency evaluated by *beta*-*actin* real-time qPCR on samples for *HLCS* and rs6636-C analysis.

|  | | | | |
| --- | --- | --- | --- | --- |
| Sample  type | Sample | Copies per reaction | | % digested |
|  |  | Mock | *Bst*UI |  |
| 1T.euploid | V0057 | 1845 | 5 | 99.7 |
| 1T.euploid | V0360 | 856 | 6 | 99.3 |
| 1T.euploid | V3104 | 1852 | 8 | 99.5 |
| 3T.euploid | PLN74 | 2937 | 5 | 99.8 |
| 3T.euploid | N0425 | 4845 | 24 | 99.5 |
| 3T.euploid | N0426 | 4283 | 33 | 99.2 |
| 1T.T21 | N0456 | 1022 | 3 | 99.7 |
| 1T.T21 | N0891 | 1315 | 13 | 99.0 |
| 1T.T21 | N3634 | 2275 | 15 | 99.3 |
| 2T.T21 | N1519 | 1607 | 28 | 98.3 |
| 3T.euploid | N2541 | 3974 | 58 | 98.5 |

1T, first trimester; 2T, second trimester; 3T, third trimester; T21, trisomy 21.
