# Supplementary material for: Epigenetic-Genetic Chromosome Dosage Approach for Fetal Trisomy 21 Detection Using an Autosomal Genetic Reference Marker
Source: PLoS One. 2010 Dec 20;5(12):e15244. doi: 10.1371/journal.pone.0015244 (PMC3004793; doi:10.1371/journal.pone.0015244)
Supplement: Table S3 — Digestion efficiency evaluated by beta-actin real-time qPCR on samples for HLCS and s6636-G analysis. (DOC) [file pone.0015244.s003.doc]

**Table S3. Digestion efficiency evaluated by *beta*-*actin* real-time qPCR on samples for *HLCS* and s6636-G analysis.**

|  | | | | |
| --- | --- | --- | --- | --- |
| Sample type | Sample | Copy per reaction | | % digested |
|  |  | Mock | *Bst*UI |  |
| 1T.euploid | V0207 | 131 | 1 | 98.9 |
| 1T.euploid | V0208 | 200 | 7 | 96.8 |
| 1T.euploid | V0284 | 856 | 2 | 99.8 |
| 1T.euploid | V0488 | 220 | 2 | 99.3 |
| 1T.euploid | V0492 | 560 | 7 | 98.8 |
| 1T.euploid | V0503 | 1036 | 2 | 99.8 |
| 1T.euploid | V0580 | 837 | 7 | 99.2 |
| 1T.euploid | V3031 | 2154 | 13 | 99.4 |
| 3T.euploid | N0069 | 4977 | 17 | 99.7 |
| 3T.euploid | PLN113 | 1873 | 31 | 98.3 |
| 3T.euploid | PLN114 | 3569 | 15 | 99.6 |
| 3T.euploid | N0333 | 2982 | 10 | 99.7 |
| 3T.euploid | N0524 | 5583 | 18 | 99.7 |
| 3T.euploid | N0527 | 5667 | 14 | 99.8 |
| 1T.T21 | N3228 | 3649 | 26 | 99.3 |
| 1T.T21 | N3438 | 3136 | 20 | 99.4 |
| 1T.T21 | N4101 | 2748 | 16 | 99.4 |
| 2T.T21 | N2913 | 2822 | 19 | 99.3 |
| 2T.T21 | N4183 | 3544 | 9 | 99.7 |
| 3T.euploid | N2582 | 2341 | 12 | 99.5 |

1T, first trimester; 2T, second trimester; 3T, third trimester; T21, trisomy 21.
